# St. Luke's Hospital

**Published:** 1854-04-01

**Authors:** 


### ST. LUKE'S HOSPITAL.

A meeting of the governors of this institution was held on Friday last, at the George and Vulture Tavern, City, for the purpose of receiving the resignation of Dr. Philp, formerly proprietor of Kensington House Lunatic Asylum, and for many years one of the visiting physicians of this hospital. Several gentlemen expressed their regret at the retirement of Dr. Philp, to whom a cordial vote of thanks was passed for the unremitting attention, ability, and zeal with which he had discharged the duties of physician to the hospital—an office he had held for twelve years. The 21st of April was fixed for electing a successor. The candidates at present announced are Dr. H. Munro, and Dr. Parker, of the London Hospital, and Dr. Arlidge.

